# Evaluation of frequency, severity, and independent risk factors for recurrence of disease activity after fingolimod discontinuation in a large real-world cohort of patients with multiple sclerosis

**DOI:** 10.1177/17562864221150312

**Published:** 2023-02-06

**Authors:** Nuria Cerdá-Fuertes, Sara Nagy, Sabine Schaedelin, Tim Sinnecker, Esther Ruberte, Athina Papadopoulou, Jens Würfel, Jens Kuhle, Özgür Yaldizli, Ludwig Kappos, Tobias Derfuss, Bernhard F. Décard

**Affiliations:** Neurology Clinic and Policlinic, Departments of Head, Spine and Neuromedicine, Biomedicine and Clinical Research, University Hospital Basel and University of Basel, Basel, Switzerland; Translational Imaging in Neurology (ThINK) Basel, Department of Medicine and Biomedical Engineering, University Hospital Basel and University of Basel, Basel, Switzerland; Neurology Clinic and Policlinic, Departments of Head, Spine and Neuromedicine, Biomedicine and Clinical Research, University Hospital Basel and University of Basel, Basel, Switzerland; Research Center for Clinical Neuroimmunology and Neuroscience (RC2NB), University Hospital Basel and University of Basel, Basel, Switzerland; Clinical Trial Unit, Department of Clinical Research, University Hospital Basel and University of Basel, Basel, Switzerland; Neurology Clinic and Policlinic, Departments of Head, Spine and Neuromedicine, Biomedicine and Clinical Research, University Hospital Basel and University of Basel, Basel, Switzerland; Medical Image Analysis Center (MIAC AG), Basel and qbig, Department of Biomedical Engineering, University of Basel, Basel, Switzerland; Medical Image Analysis Center (MIAC AG), Basel and qbig, Department of Biomedical Engineering, University of Basel, Basel, Switzerland; Translational Imaging in Neurology (ThINK) Basel, Department of Medicine and Biomedical Engineering, University Hospital Basel and University of Basel, Basel, Switzerland; Neurology Clinic and Policlinic, Departments of Head, Spine and Neuromedicine, Biomedicine and Clinical Research, University Hospital Basel and University of Basel, Basel, Switzerland; Translational Imaging in Neurology (ThINK) Basel, Department of Medicine and Biomedical Engineering, University Hospital Basel and University of Basel, Basel, Switzerland; Medical Image Analysis Center (MIAC AG), Basel and qbig, Department of Biomedical Engineering, University of Basel, Basel, Switzerland; Neurology Clinic and Policlinic, Departments of Head, Spine and Neuromedicine, Biomedicine and Clinical Research, University Hospital Basel and University of Basel, Basel, Switzerland; Research Center for Clinical Neuroimmunology and Neuroscience (RC2NB), University Hospital Basel and University of Basel, Basel, Switzerland; Neurology Clinic and Policlinic, Departments of Head, Spine and Neuromedicine, Biomedicine and Clinical Research, University Hospital Basel and University of Basel, Basel, Switzerland; Translational Imaging in Neurology (ThINK) Basel, Department of Medicine and Biomedical Engineering, University Hospital Basel and University of Basel, Basel, Switzerland; Neurology Clinic and Policlinic, Departments of Head, Spine and Neuromedicine, Biomedicine and Clinical Research, University Hospital Basel and University of Basel, Basel, Switzerland; Research Center for Clinical Neuroimmunology and Neuroscience (RC2NB), University Hospital Basel and University of Basel, Basel, Switzerland; Neurology Clinic and Policlinic, Departments of Head, Spine and Neuromedicine, Biomedicine and Clinical Research, University Hospital Basel and University of Basel, Basel, Switzerland; Research Center for Clinical Neuroimmunology and Neuroscience (RC2NB), University Hospital Basel and University of Basel, Basel, Switzerland; Neurology Clinic and Policlinic, Departments of Head, Spine and Neuromedicine, Biomedicine and Clinical Research, University Hospital Basel and University of Basel, Basel, Switzerland

**Keywords:** discontinuation, disease activity, fingolimod, multiple sclerosis, rebound, switch

## Abstract

**Background:**

Clinical and radiological signs of recurring disease activity (RDA) have been described in patients with multiple sclerosis (pwMS) after discontinuation of fingolimod (FGL).

**Objective:**

To describe frequency, severity and potential risk factors for RDA after FGL discontinuation in a large real-world cohort of pwMS.

**Methods:**

Post-FGL RDA was defined as evidence of clinical and/or radiological activity within 6 months after FGL discontinuation. Relapses with Expanded Disability Status Scale increase ⩾2 points and/or magnetic resonance imaging (MRI) activity with at least five cerebral gadolinium-enhancing lesions and/or ⩾6 cerebral new T2 lesions were defined as severe recurring disease activity (sRDA). Using a multivariate logistic model, we explored the influence of age, disease duration, sex, clinical, and MRI activity under FGL on the occurrence of RDA.

**Results:**

We identified 110 pwMS who discontinued FGL. Thirty-seven (33.6%) developed post-FGL RDA and 13 (11.8%) also fulfilled criteria for sRDA. Younger age at diagnosis [odds ratio (OR) = 1.10, *p* < 0.01], shorter disease duration (OR = 1.17, *p* < 0.01), and MRI activity under FGL (OR = 2.92, *p* = 0.046) were independent risk factors for the occurrence of post-FGL RDA.

**Conclusion:**

Individual risk assessment and optimal treatment sequencing can help to minimize the risk of post-FGL RDA. Early switch to highly effective disease-modifying therapy might reduce occurrence of post-FGL RDA.

## Introduction

Fingolimod (FGL) was the first oral disease-modifying therapy (DMT) approved for the treatment of relapsing-remitting multiple sclerosis (RRMS) and is available as first-line treatment in Switzerland since 2011.

In recent years, an increasing number of case reports^1–7^ as well as retrospective cohort studies reported the occurrence of recurring disease activity (RDA) after FGL discontinuation.^8–15^ On the contrary, a post hoc analysis of pooled data from FGL phase III studies did not find an increased individual risk of unexpectedly high clinical or radiological RDA after FGL discontinuation compared with placebo.^16^ A recently published ‘FDA drug safety communication’, however, informed about the potential risk of severe worsening after stopping FGL.^17^

Depending on the clinical situation FGL is usually stopped without subsequent therapy (e.g. in case of childbearing preferences or pregnancy) or followed by another DMT (e.g. in case of ongoing disease activity or side effects under FGL). To date, there is only sparse real-world data concerning clinical factors or settings, which might predict the risk of RDA after FGL discontinuation.

In this retrospective study, we aimed to investigate the frequency and severity of disease activity after FGL discontinuation in a large monocentric cohort of patients with multiple sclerosis (pwMS) and identify independent risk factors for the occurrence of post-FGL RDA. Within 6 months after FGL discontinuation, we also analyzed available quantitative magnetic resonance imaging (MRI) data in a subgroup of pwMS and the repopulation of blood lymphocytes.

## Material and methods

### Clinical characteristics

**Figure 1.**
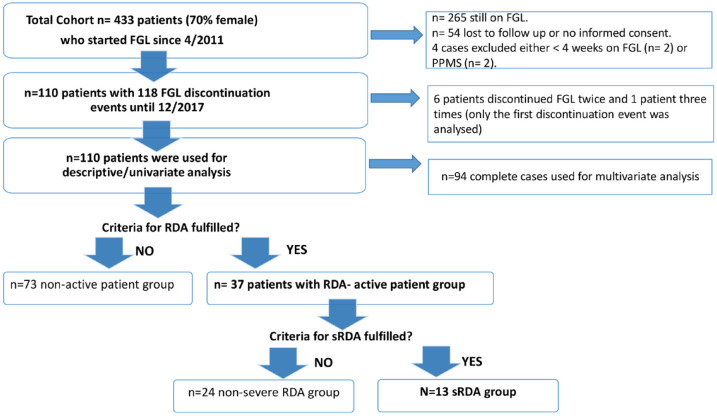
Flowchart of patients’ selection. FGL, fingolimod; PPMS, primary progressive multiple sclerosis; RDA, recurring disease activity; sRDA, severe recurring disease activity.

Medical records of pwMS who initiated FGL at the multiple sclerosis (MS) center Basel since April 2011 were screened retrospectively. For chart review and further analysis, we included only pwMS with FGL treatment duration of at least 4 weeks, who discontinued FGL until 12/2017 (switch to another DMT or stop without subsequent DMT within 6 months) with available data from regular clinical visits at least 6 months after FGL discontinuation and who signed an informed consent (Figure 1).

The inclusion criteria and following definitions (section ‘Definition of post-FGL RDA and severe recurring disease activity’) are the result of face-to-face meetings with structured communication methods among members of our MS expert group, corresponding to a ‘mini-Delphi’ process. The study was approved by the local ethics committees (Project-ID 2019-02254). Three board certified neurologists (N.C.-F., S.N., B.F.D.) with expertise in diagnosis and treatment of MS were responsible for the chart review and retrospective data collection.

For evaluation of clinical disease activity under FGL treatment, occurrence and frequency of relapses, as well as Expanded Disability Status Scale (EDSS) progression, were analyzed. MRI activity [new T1 gadolinium (Gad+) or new T2 lesion] under FGL treatment was evaluated qualitatively as ‘yes’ or ‘no’. Owing to the retrospective study design, data from standardized re-baseline MRI after FGL treatment initiation were not always available. Time point of new T2 lesion occurrence was not unequivocal in all cases. If MRI activity was not clearly attributable to the FGL treatment period, we rated these cases as ‘not attributable’. In pwMS who discontinued FGL several times, only the first discontinuation event was analyzed.

### Definition of post-FGL RDA and severe recurring disease activity

1. RDA was defined as evidence of clinical disease activity (relapses) and/or cerebral or spinal MRI activity (Gad+ enhancement) occurring within 6 months after FGL discontinuation.
2. Severe recurring disease activity (sRDA) was defined as severe relapse (EDSS increase ⩾2 points) and/or pronounced MRI activity with at least five cerebral Gad+ enhancing lesions and/or ⩾6 new cerebral T2 lesions occurring within 6 months after FGL discontinuation.

### Statistical methods

Baseline characteristics are summarized for the cohort of pwMS who discontinued FGL. Categorical variables are presented as count and frequency. For continuous variables, the mean value and the standard deviation, or in case of skewed data, the median and the interquartile range (IQR), are presented.

Then, we summarized and compared potential risk factors of pwMS with post-FGL RDA (active pwMS) and pwMS with stable post-FGL disease course (nonactive pwMS) as described above. Data were compared using chi-square tests and *t* tests. In case of violation of the normal assumption, the Wilcoxon rank sum tests were used.

The association between RDA and potential risk factors was assessed in logistic regression models. Sex, age at diagnosis, disease duration, disease activity under FGL [quantified as annualized relapse rate (ARR), MRI activity under FGL (qualitatively evaluated as ‘yes’ or ‘no’), and EDSS progression], duration of FGL, treatment before FGL, and lymphopenia under FGL were predefined as potential risk factors for RDA. Starting from these variables, we performed backward selection based on the Akaike information criterion (AIC) in order to find the variables strongest associated with RDA. The resulting model was presented as odds ratios together with its confidence interval based on likelihood profiling as well as a *p* value (likelihood ratio test). Variable selection and multivariate analysis were performed on complete cases only (*n* = 94). Baseline characteristics in pwMS with missing values were compared with complete cases in order to assess if the complete cases are representative for the whole population.

We also analyzed the DMT started after FGL stop and the time point of their initiation (switch interval). Owing to the retrospective study design, however, we had to deal with unstandardized switch intervals, small sample size of different post-FGL treatment groups, and different FGL switch reasons (e.g. disease activity under FGL or childbearing preferences). In order to overcome statistical problems of small sample sizes in the post-FGL treatment groups, we defined clinically relevant post-FGL treatment subgroups [platform DMT, oral DMT, and highly effective disease-modifying therapy (heDMT)]. For statistical reasons (e.g. immortal time bias), reliable analyses to study the effect of switch intervals and DMT on post-FGL RDA are not feasible with the available retrospective data. In order to at least estimate the effect of DMT and switch interval in our data set, we decided to analyze two extreme switch interval subgroups (switch to another DMT within 8 weeks or after 8 weeks); in this scenario, we assumed that pwMS who started a DMT >8 weeks after FGL discontinuation behave like pwMS who stopped FGL without subsequent DMT concerning the post-FGL RDA.

Then, we compared the previously identified risk factors for RDA in two extreme groups: (1) pwMS that were more than 8 weeks without a succeeding DMT (excluding women who discontinued due to pregnancy or childbearing preferences) and (2) pwMS that switched to an heDMT in less than 8 weeks (excluding those who had a relapse prior to the initiation of a subsequent DMT).

Furthermore, we evaluated lymphocyte counts in pwMS with and without RDA under FGL and after discontinuation. Using univariate statistical analyses within the subgroup of RDA, we also compared potential risk factors of pwMS with nonsevere RDA and sRDA. Finally, MRI parameters were summarized for these two groups as median and interquartile range (lesion volume and count) or as mean value and standard deviation (mean volume per lesion).

### Quantitative MRI analysis after FGL discontinuation in subgroup of pwMS with post-FGL RDA

Last cerebral MRI available before (baseline scan) and follow-up cerebral MRIs available within 6 months after FGL cessation were analyzed quantitatively [median (IQR) time to FGL stop 71 days (22.5–183), median (IQR) time after FGL stop 101 days (52.25–136.25)]. A total of 36 baseline and 27 follow-up cerebral MRIs were available. T2 lesions from three follow-up MRIs could not be analyzed due to insufficient data quality or missing sequences. If the time from baseline MRI to FGL discontinuation was less than 2 months, new T2 lesions in follow-up MRIs were attributed to the period without FGL.

MRI data were analyzed at the Medical Imaging Center in Basel (MIAC). One patient who had tumefactive lesions in the last cerebral MRI scan under FGL treatment was excluded from this analysis. MRI protocol included a pre- and post-contrast T1-weighted (T1w), and a two-dimensional (2D) axial dual echo proton density and T2-weighted (T2w) sequence with turbo spin echo read-out. Tree-dimensional (3D) T1w images were co-registered to 2D T2w images. T2w sequences were generated without contrast. Images were manually analyzed by experienced investigators (T.S., E.R., J.W.) in consensus reading using Amira (Mercury Computer Systems Inc., Chelmsford, MA, USA). The number and volume of T2w hyperintense lesions and of Gad+ enhancing lesions on postcontrast T1w images were analyzed at baseline and follow-up. New T2w lesions were defined as focal hyperintense signal alterations of five or more voxels on precontrast 2D axial dual echo proton density and T2w sequence that were not present on the reference scan. Perivascular spaces were excluded by their tubular appearance.

### Lymphocyte repopulation

Baseline lymphocyte counts under FGL (first available lymphocyte count after FGL start) and 1, 3, and 6 months after FGL discontinuation were collected. Baseline counts were available in 108 pwMS, while counts 1, 3, and 6 months after discontinuation were available in 67, 69, and 76 pwMS, respectively.

## Results

### Descriptive analysis and clinical baseline characteristics

We identified 433 pwMS (70% female) who started FGL since April 2011, among them, 110 pwMS discontinued the treatment (see Figure 1). Baseline characteristics of these 110 pwMS are summarized in Table 1. Approximately half of the pwMS were on FGL based on an escalation approach after prior platform therapies. As FGL is a first-line therapy in Switzerland, 19% were therapy naïve, 23% de-escalated from natalizumab to FGL, and 5% had other previous therapies.

**Table 1.** Baseline characteristics of the study population.

	Overall
*n*	110
Sex = female (%)	79 (71.8)
Disease course at discontinuation RRMS (%)	91 (82.7)
Age at diagnosis [mean (SD)]	30.65 (9.87)
Age at discontinuation [mean (SD)]	40.22 (11.18)
Disease duration at discontinuation (years) (median [IQR])	9.50 [3.00–13.75]
EDSS at FGL start (median [IQR])	3.0 [2.0–4.0]
Duration on FGL (months) [mean (SD)]	26.98 (19.12)
ARR under FGL (median [IQR])	0.00 [0.00–0.77]
Previous DMT (%)	
Platform	−58 (52.7)
NTZ	−25 (22.7)
Others	−6 (5.5)
Treatment naïve	−21 (19.1)
Time to next DMT more than 8 weeks (%)	49 (44.5)
New DMT within 8 weeks^a^ (%)	
heDMT	−39 (35.5)
Orals	−12 (10.9)
Platform	−6 (5.5)
Reactive treatment^b^ (%)	4 (3.6)
Reason for discontinuation (%)	
Pregnancy/childbearing preferences	−16 (14.5)
Disease activity	−48 (43.6)
Side effects	−24 (21.8)
Noncompliance	−2 (1.8)
Others	−20 (18.2)

ARR, annualized relapse rate; DMT, disease-modifying therapy; EDSS, Expanded Disability Status Scale; FGL, fingolimod; heDMT, highly effective disease-modifying therapy; IQR, interquartile range; NTZ, natalizumab; RRMS, relapsing-remitting multiple sclerosis.

a New DMT started within 8 weeks after FGL discontinuation are summarized in the table and divided in highly effective disease-modifying therapies (natalizumab, ocrelizumab, rituximab), orals (teriflunomide, azathioprine, dimethyl fumarate) and platform (interferon beta, glatiramer acetate). In the group of patients who did not start a new DMT within 8 weeks, we have not summarized the next treatment.

b Finally, four pwMS had a relapse during the treatment-free period and started another DMT as a consequence of this new disease activity, this group is presented separately as reactive treatment.

Most frequent reasons for FGL discontinuation were (1) disease activity under treatment (44%), (2) side effects (22%), and (3) childbearing preferences or pregnancy (14%). Other reasons included patient’s own preferences and concomitant diagnosis (e.g. tumor, rheumatoid arthritis). A total of 66 pwMS switched to another DMT within 6 months and 57 pwMS (52%) re-started a DMT within 8 weeks after FGL stop, 49 pwMS (44%) were more than 8 weeks without DMT. Four pwMS had a relapse during the treatment-free period and started another DMT as a consequence of this new disease activity.

### Occurrence of post-FGL RDA and associated risk factors

Thirty-seven pwMS (30 female) fulfilled criteria for RDA within 6 months after FGL discontinuation. Nineteen pwMS (51%) had clinical and MRI activity (3 only spinal MRI activity, but not cerebral), 13 pwMS (35%) had clinical activity only (of which 2 had a relapse without new T2 or Gad+ enhancing lesions on MRI, the rest did not receive an MRI or had new T2 lesions that could not be attributed to the period without FGL), and 5 pwMS (14%) had isolated MRI activity. In pwMS with clinical post-FGL RDA, median time to relapse was 8 weeks (IQR 4-16 weeks).

Descriptive, univariate statistical analysis comparing pwMS with stable post-FGL disease course (nonactive pwMS) and pwMS with post-FGL RDA (active pwMS) is summarized in Table 2.

**Table 2.** Univariate statistical analysis comparing patients with postfingolimod recurring disease activity (active) and patients without postfingolimod recurring disease activity (nonactive).

	Active	Nonactive	*p*
*n*	37	73	
Sex = female (%)	30 (81.1)	49 (67.1)	0.189
Age at diagnosis [mean (SD)]	26.62 (7.51)	32.68 (10.34)	0.002
Age at discontinuation [mean (SD)]	33.32 (9.06)	43.71 (10.56)	<0.001
Disease duration^a^ (years) (median [IQR])	5 [3.00–10.00]	11 [5.00–15.00]	0.003
ARR under FGL [mean (SD)]	0.58 (0.94)	0.55 (1.10)	0.883
EDSS progression under FGL^b^ [mean (SD)]	0.36 (1.19)	0.34 (1.01)	0.939
MRI activity under FGL = yes (%)	23 (71.9)	29 (43.9)	0.017
Duration on FGL months [mean (SD)]	24.11 (16.66)	28.44 (20.20)	0.264
Previous DMT (%)			
Platform	−21 (56.8)	−37 (50.7)	
NTZ	−9 (24.3)	−16 (21.9)	
Others	−0 (0.0)	−6 (8.2)	
Treatment naïve	−7 (18.9)	−14 (19.2)	
Lymphopenia on FGL = yes (%)^c^	35 (94.6)	70 (98.6)	0.560
Lymphocyte count 1 month (×10^9^/l) (median [IQR])	1.17 [0.94–1.29]	1.07 [0.77–1.42]	0.617
Lymphocyte count 3 months (×10^9^/l) (median [IQR])	1.05 [0.87–1.42 ]	1.15 [0.76–1.47]	0.985
Lymphocyte count 6 months (×10^9^/l) (median [IQR])	1.29 [0.90–1.66 ]	1.10 [0.89–1.43]	0.373
Time to next DMT more than 8 weeks (%)	18 (48.6)	31 (42.5)	
New DMT within 8 weeks^d^ (%)			
heDMT	−9 (24.3)	−30 (41.1)	
Orals	−1 (2.7)	−11 (15.1)	
Platform	−5 (13.5)	−1 (1.4)	
Reactive treatment^e^ (%)	4 (10.8)	0 (0.0)	

ARR, annualized relapse rate; DMT, disease-modifying therapy; EDSS, Expanded Disability Status Scale; FGL, fingolimod; heDMT, highly effective disease-modifying therapy; IQR, interquartile range; MRI, magnetic resonance imaging; NTZ: natalizumab.

a Note that disease duration was calculated at fingolimod discontinuation.

b EDSS progression under FGL: an increase on the EDSS of at least 0.5 points for EDSS at fingolimod start ⩾5.5, and of at least 1 point if EDSS at fingolimod start <5.5.

c Lymphopenia was defined as ⩽0.9 × 10^9^/l.

d New DMT started within 8 weeks after FGL discontinuation are summarized in the table and divided in highly effective disease-modifying therapy (natalizumab, ocrelizumab, rituximab), orals (teriflunomide, azathioprine, dimethyl fumarate), and platform (interferon beta, glatiramer acetate).

e Highly effective disease-modifying therapy in each group: active group (6 rituximab and 3 natalizumab, in addition, 4 patients had a relapse during the treatment-free period and started rituximab as a consequence of this new disease activity, this last group is presented separately, as reactive treatment) and nonactive group (21 rituximab, 7 natalizumab, 2 ocrelizumab).

A total of 94 completed cases were used for the multivariate logistic model (Figure 1) to explore the influence of age, disease duration, sex, and clinical and MRI activity under FGL on the occurrence of RDA. Of note, our complete cases population was representative for the whole cohort (data not shown). Using a multivariate logistic model, we were able to demonstrate that younger age at diagnosis [effect of 1 year less odds ratio (OR) = 1.098, *p* < 0.01], shorter disease duration (effect of 1 year less OR = 1.167, *p* < 0.01), and MRI activity under FGL treatment (OR = 2.916, *p* = 0.046) represent independent risk factors for the occurrence of post-FGL RDA (Table 3).

**Table 3.** Independent risk factors for postfingolimod disease activity.

	Coefficient	SD	OR	CI	*p* value	*N*
(Intercept)	2.788	1.190	16.251	[1.730, 193.338]	NA	94
Age at diagnosis (effect of 1 year less)	0.094	0.033	1.098	[1.033, 1.179]	<0.01	VIF = 1.12
Disease duration^a^ (effect of 1 year less)	0.154	0.046	1.167	[1.074, 1.287]	<0.01	VIF = 1.17
MRI activity under FGL present (*versus* absent)	1.070	0.550	2.916	[1.018, 8.982]	0.0462	VIF = 1.09

CI, confidence interval; FGL, fingolimod; NA, not available; OR, odds ratio; SD, standard deviation; VIF, variance inflation factor.

Multivariate logistic model with 94 complete cases that shows that younger age at diagnosis, shorter disease duration, and presence of MRI activity under FGL are independent risk factors for postfingolimod disease activity.

a Note that disease duration was calculated at fingolimod discontinuation.

pwMS who switched to heDMT within 8 weeks had more frequently MRI activity under FGL. We, however, did not observe a higher post-FGL RDA compared with pwMS who were more than 8 weeks without a subsequent DMT (Table 4).

**Table 4.** Comparison of risk factors for recurring disease activity by switch interval and subsequent therapy.

	More than 8 weeks pause	Less than 8 weeks, heDMT	*p*
*n*	38	39	
Nonactive (%)	26 (68.4)	30 (76.9)	0.561
Age at diagnosis [mean (SD)]	32.82 (9.20)	29.41 (8.73)	0.100
Disease duration^a^ (median [IQR])	10.00 [2.25–13.00]	11.00 [7.00–14.00]	0.397
MRI activity under FGL = yes^b^ (%)	9 (30.0)	27 (71.1)	0.002

FGL, fingolimod; heDMT, highly effective disease-modifying therapy; IQR, interquartile range; MRI, magnetic resonance imaging; SD, standard deviation.

This table shows a comparison of the previously identified risk factors for recurring disease activity in two extreme groups: (1) patients who were more than 8 weeks without a succeeding DMT (excluding those women who discontinued due to pregnancy or childbearing preferences) and (2) patients who switched to an heDMT in less than 8 weeks (excluding those who had a relapse prior to the initiation of a subsequent DMT).

a Note that disease duration was calculated at fingolimod discontinuation.

b In nine patients (eight in ‘more than 8 weeks pause’ group and one in ‘less than 8 weeks, heDMT’ group), MRI activity under FGL could be not be determined. These patients are ignored in the variable ‘MRI activity under FGL’. We performed a sensitivity analysis, and the results remained unchanged (data not shown).

**Figure 2.**
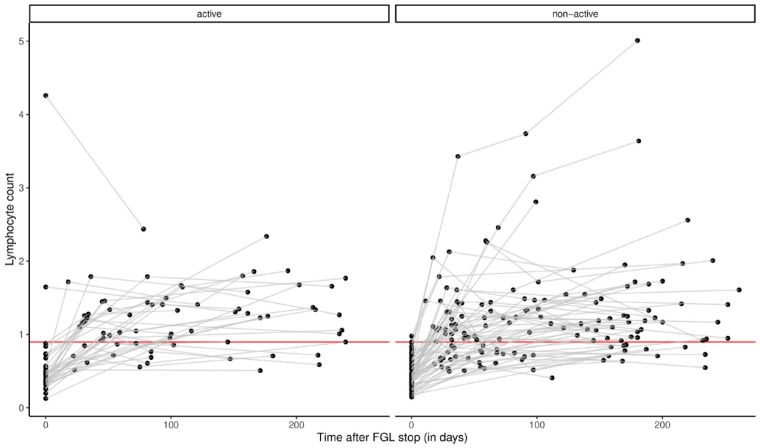
Lymphocyte counts after fingolimod discontinuation in active and nonactive group. Y-axis represents the lymphocyte counts (×10^9^/l). X-axis represents the time after FGL stop in days. The values at time = 0 indicate the first lymphocyte counts after FGL start. The red line indicates the lowest lymphocyte normal count (for statistical comparison, see Table 2). FGL, fingolimod.

Analysis of baseline lymphocyte counts and lymphocyte repopulation after FGL discontinuation did not show differences between active and nonactive pwMS (Figure 2 and Table 2).

### Subgroup analysis in pwMS with post-FGL RDA

#### Occurrence of post-FGL sRDA

sRDA within 6 months after FGL discontinuation was observed in 13 pwMS (10 female, 3 male). Four pwMS (≈4% of pwMS who discontinued FGL) had a confirmed disability worsening ⩾1 EDSS point after a median follow-up of 6 months since post-FGL relapse. pwMS who developed sRDA had similar demographic and clinical characteristics than those with nonsevere RDA (Table 5). The majority of pwMS with sRDA (77%) had a relapse in the first 8 weeks after FGL discontinuation, while in the nonsevere RDA group, half of the pwMS developed a relapse between 8 and 24 weeks after FGL discontinuation. There were no differences in lymphocyte levels 1, 3, and 6 months after FGL discontinuation between both groups.

**Table 5.** Univariate statistical analysis comparing patients with nonsevere and severe recurring disease activity.

	Nonsevere	Severe	*p*
*n*	24	13	
Sex = female (%)	20 (83.3)	10 (76.9)	0.972
Age at diagnosis [mean (SD)]	26.83 (6.91)	26.23 (8.81)	0.820
Age at discontinuation [mean (SD)]	32.92 (8.54)	34.08 (10.27)	0.715
Disease duration^a^ (years) (median [IQR])	4.00 [2.75–9.25]	6 [4.00–10.00]	0.406
ARR under FGL [mean (SD)]	0.47 (0.69)	0.78 (1.29)	0.354
EDSS progression under FGL^b^ [mean (SD)]	0.54 (1.30)	0.00 (0.88)	0.205
MRI activity under FGL = yes (%)	14 (70.0)	9 (75.0)	1.000
Duration on FGL (months) [mean (SD)]	20.67 (16.58)	30.46 (15.44)	0.088
Previous DMT (%)			
Platform	−13 (54.2)	−8 (61.5)	
NTZ	−6 (25.0)	−3 (23.1)	
Treatment naïve	−5 (20.8)	−2 (15.4)	
Lymphopenia on FGL = yes (%)^c^	22 (91.7)	13 (100.0)	0.758
Lymphocyte count 1 month (×10^9^/l) (median [IQR])	1.19 [0.95–1.34]	1.16 [1.02–1.21]	0.721
Lymphocyte count 3 months (×10^9^/l) (median [IQR])	1.05 [0.86–1.41]	1.05 [0.88–1.44]	1.000
Lymphocyte count 6 months (×10^9^/l) (median [IQR])	1.31 [0.98–1.60]	1.29 [0.90–1.66]	0.887
Time to next DMT more than 8 weeks (%)	15 (62.5)	3 (23.1)	
New DMT within 8 weeks^d^ (%)			
heDMT	−4 (16.7)	−5 (38.5)	
Orals	−0 (0.0)	−1 (7.7)	
Platform	−2 (8.3)	−3 (23.1)	
Reactive treatment (%)	3 (12.5)	1 (7.7)	
Time until relapse categorized (%)			
No relapse	−4 (16.7)	−1 (7.7)	
0–8 weeks	−8 (33.3)	−10 (76.9)	
8–24 weeks	−12 (50.0)	−2 (15.4)	
Time until relapse (weeks)^e^ (median [IQR])	12 [4.00–16.00]	8.00 [4.00–8.00]	0.114
Any baseline T1 CE lesion = yes (%)	3 (13.0)	3 (23.1)	0.756
Mean volume per T1 CE lesion at baseline (mm^3^) [mean (SD)]	3.42 (9.75)	32.58 (68.39)	0.050
T2 lesion count at baseline (median [IQR])	23.00 [13.00–42.00]	67.00 [38.00–95.50]	0.006
T2 lesion count norm at baseline^f^ (median [IQR])	5.75 [1.98–11.92]	8.00 [4.59–16.66]	0.094
T2 lesion volume at baseline (mm^3^) (median [IQR])	2511.00 [837.70–5412.95]	6454.80 [3796.70–9610.95]	0.034
T2 lesion volume norm at baseline^f^ (mm^3^) (median [IQR])	425.47 [191.15–1242.51]	891.85 [506.32–1654.52]	0.094
Mean volume per T2 lesion at baseline (mm^3^) [mean (SD)]	103.19 (59.73)	126.20 (79.06)	0.351

ARR, annualized relapse rate; CE, contrast enhancing; DMT, disease-modifying therapy; EDSS, Expanded Disability Status Scale; FGL, fingolimod; NTZ, natalizumab; norm, normalized.

Summary and group comparison of quantitative MRI data at baseline are shown. Baseline scan is last cerebral MRI available before FGL cessation [median (IQR) time to FGL stop 71 days (22.5–183)].

a Note that disease duration was calculated at fingolimod discontinuation.

b EDSS progression under FGL: an increase in the EDSS of at least 0.5 points for EDSS at fingolimod start ⩾5.5, and of at least 1 point if EDSS at fingolimod start <5.5.

c Lymphopenia was defined as ⩽0.9 × 10^9^/l.

d New treatments started within 8 weeks after fingolimod discontinuation are summarized in the table and divided in highly effective disease-modifying therapies (natalizumab, ocrelizumab, rituximab), orals (teriflunomide, azathioprine, dimethyl fumarate) and platform (interferon beta, glatiramer acetate). Highly effective disease-modifying therapies in each group: nonsevere group (3 rituximab, 1 natalizumab) and nonsevere (3 rituximab, 2 natalizumab).

e Note that in the variable ‘time until relapse’, only the patients who had a relapse are included.

f T2 lesion count and volume have been normalized for disease duration.

#### Quantitative MRI analysis in subgroup of pwMS with RDA

pwMS with sRDA had a higher mean volume per T1 Gad+ lesion at baseline MRI before FGL discontinuation (32.58 *versus* 3.42 mm^3^). Furthermore, they had a higher T2 lesion count and T2 lesion volume at baseline than pwMS with nonsevere post-FGL RDA (67 *versus* 23 T2 lesions and 6454.80 *versus* 2511.00 mm^3^, respectively); however, there was no difference after normalization for disease duration (Table 5).

At follow-up, MRI scans from pwMS with sRDA had a higher median volume per new T2 lesion (209.5 *versus* 76.5 mm^3^) than scans from pwMS with nonsevere post-FGL RDA (Table 6).

**Table 6.** Descriptive MRI analysis of follow-up scan in active patients (severe *versus* nonsevere postfingolimod disease activity).

Endpoint	Group	*N*	Median	IQR	NAs
T2 new lesion number at FU	Severe	10	9	[8–15]	3
T2 new lesion number at FU	Nonsevere	14	2.5	[1–4]	10
T2 new lesion volume at FU (mm^3^)	Severe	10	2070.25	[1027.075–2783.25]	3
T2 new lesion volume at FU (mm^3^)	Nonsevere	14	193.45	[79.025–448.825]	10
Median volume per T2 new lesion at FU (mm^3^)	Severe	10	209.5	[104.75–234]	3
Median volume per T2 new lesion at FU (mm^3^)	Nonsevere	14	76.5	[58.25–111.25]	10

FGL, fingolimod; FU, follow-up; IQR, interquartile range; MRI, magnetic resonance imaging; NA, not available.

Follow-up cerebral MRIs available within 6 months after FGL cessation were analyzed quantitatively [median (IQR) time after FGL stop 101 days (52.25–136.25)].

## Discussion

In our cohort of 110 pwMS discontinuing FGL, 37 pwMS (34%) developed RDA within 6 months after FGL cessation. Of those, 13 pwMS (12% of overall group) were considered to have sRDA and 4 pwMS (4% of overall group) had a confirmed disability worsening ⩾1 EDSS point after a median follow-up of 6 months since post-FGL relapse. The frequency of post-FGL RDA in our cohort is similar as previously reported in the literature.^8–15^

**Figure 3.**
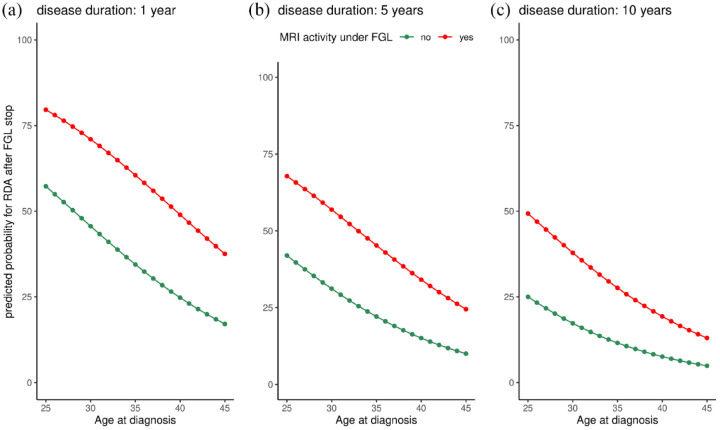
Predicted probability for recurring disease activity. Predictive probability for recurring disease activity (Y-axis) in patients with a disease duration of 1 year (a), 5 years (b), and 10 years (c) depending on age at diagnosis (X-axis) and the presence of MRI activity under FGL (red line indicates the presence of MRI activity under FGL and green line indicates the absence of MRI activity under FGL). FGL, fingolimod.

Using a multivariate statistical approach, this study demonstrates that younger age at diagnosis, shorter disease duration, and evidence of MRI activity under FGL are independent risk factors for the occurrence of post-FGL RDA. Figure 3 illustrates the influence of these independent risk factors on the probability of RDA and allows a personalized risk assessment for the occurrence of RDA in clinical practice. To the best of our knowledge, no other published study has performed multivariate analysis to determine clinically relevant independent risk factors for the occurrence of post-FGL disease activity.

In an Italian cohort,^9^ pwMS with post-FGL sRDA were all females and younger than the other subjects; however, these differences were not statistically significant. In contrast to our study, pwMS who discontinued FGL due to inefficacy were excluded in this study by Frau *et al.*^9^ As the presence of disease activity under FGL is a common reason for treatment discontinuation in clinical practice (in our cohort 44%) and consecutive switch to heDMT, it is important to investigate this subgroup. Our study demonstrated that the presence of MRI activity under FGL even represents an independent risk factor for the occurrence of post-FGL RDA. The results of our quantitative analyses also suggest that risk for post-FGL RDA seems to be elevated in a subgroup of pwMS with higher baseline MRI disease activity.

Another recently Italian cohort with 230 pwMS, including those who discontinued due to inefficacy,^14^ found that a higher age at FGL discontinuation was associated with a lower probability of sRDA. In our cohort, pwMS without RDA were also older than pwMS with RDA at FGL discontinuation (Table 2); however, this difference did not remain significant in the multivariate logistic model. Pantazou *et al.*^15^ demonstrated that the incidence of RDA is not negligible in pwMS over 50-year-old; nevertheless, the incidence of RDA in this study was half less in the older pwMS.

Owing to statistical reasons, this retrospective cohort study was not suitable to investigate the effect of switch interval and subsequent DMT selection on the occurrence of post-FGL RDA. In contrast to the other published cohort studies who investigated the phenomena of post-FGL RDA, however, we tried to overcome statistical shortcomings and estimated the effect of early switch to heDMT using two extreme scenarios. It was not surprising that pwMS who switched to heDMT within 8 weeks post-FGL had more frequently MRI activity under FGL than the other group (Table 4). This observation in our retrospective data set is certainly a consequence of an intentional decision-making process of the treating physician. Although these pwMS with evidence of MRI activity under FGL have a higher risk for RDA, they showed more frequently a stable post-FGL disease course. Furthermore, most of our pwMS in the sRDA group had a relapse in 8 weeks or less after FGL discontinuation. These results have a hypothesis generating character and could be of great importance for the clinical management in the future: in pwMS with evidence of MRI activity under FGL, a fast switch to an heDMT should be considered to reduce the risk of post-FGL RDA. Prospective studies with standardized switching protocols are needed to further investigate the effect of switch intervals and DMT selection on the occurrence of post-FGL RDA.

In a Turkish cohort with 31 pwMS, sRDA was more likely in pwMS with lower EDSS scores and higher ARR before starting FGL.^13^ These observations are also in line with our results, indicating that pwMS who have a highly active disease course are at risk for post-FGL RDA. In clinical practice, physicians are confronted with long disease trajectories, incomplete data access, sequential DMT, and short switch intervals. Thus, assessment of pre-FGL disease activity in real-life is often difficult. The term ‘rebound’, however, has been defined as RDA following the discontinuation of natalizumab that exceeds levels of pretreatment disease activity.^18^ Several terms have been used to describe this phenomenon after FGL discontinuation, such as ‘severe disease reactivation’,^9,13,14^ ‘disease exacerbation’,^11^ ‘outliers’^16^, and ‘rebound’.^8,10,12^ In our cohort, approximately 20% of pwMS were treatment naïve and started FGL right after disease diagnosis and another 23% received natalizumab prior to FGL. In both groups, calculation of pre-FGL ARR is neither useful nor meaningful in clinical practice. For this reason, we avoided the terms ‘rebound’ or ‘disease reactivation’. In contrast to previously published studies, we tried to establish an individual risk assessment for post-FGL RDA based on easily available clinical and demographic variables (Figure 3), which can be used for ‘every day’ counseling of pwMS.

**Figure 4.**
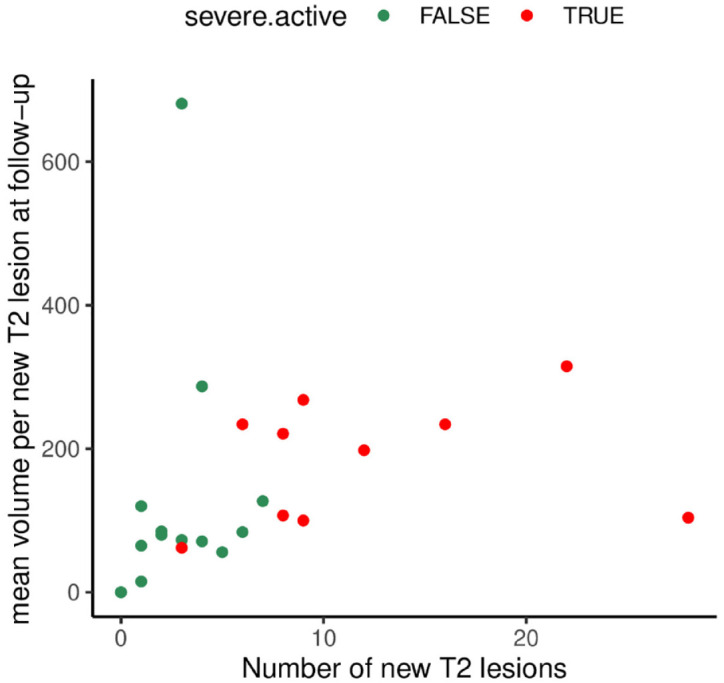
MRI patterns of active patients at follow-up. The graphic represents the mean volume per new T2 lesion at follow-up (mm^3^, Y-axis) and the number of new T2 lesions (X-axis) in patients with severe recurring disease activity (red) *versus* patients with nonsevere recurring disease activity (green). Patients with severe recurring disease activity had (per definition) a higher number of T2 lesions, but there was no specific MRI pattern detectable.

Quantitative MRI analysis in the subgroup with post-FGL RDA showed that pwMS with sRDA had a higher T2 lesion count and T2 lesion volume at baseline than those with nonsevere RDA. Although this difference was not significant after correcting for disease duration, it supports the notion that the degree of post-FGL RDA seems to be associated with the baseline disease activity.^15,16^ Lapucci *et al.*^19^ observed tumefactive and punctated MRI patterns in pwMS with post-FGL sRDA, while those with nonsevere RDA had MS typical MRI activity. In our cohort, pwMS with post-FGL sRDA had a trend to larger mean volume per new T2 lesion at follow-up scan (Figure 4); however, we could not identify a specific post-FGL MRI pattern.

The pathophysiological mechanisms causing RDA after FGL discontinuation are not completely understood. It has been hypothesized that a fast increase of circulating lymphocytes and consecutive re-entry of encephalitogenic lymphocytes into the central nervous system (CNS) may promote ‘rebound phenomena’.^8^ Standardized clinical data on lymphocyte levels at FGL discontinuation and time of repopulation after its discontinuation and its association with RDA are lacking. Available data from observational studies showed no consistent findings: in 19 pwMS who switched from FGL to dimethyl fumarate,^11^ peripheral blood lymphocyte counts at the time of FGL discontinuation were significantly lower in those pwMS with post-FGL disease exacerbation. In another cohort with 230 pwMS,^14^ those with prolonged lymphopenia had a higher risk of relapses. In our cohort, lymphocyte counts under FGL and 1, 3, and 6 months after its discontinuation did not differ between pwMS with or without post-FGL RDA.

Neuropathological descriptions from an autoptic case of fatal post-FGL rebound indicated that overexpression of S1P receptors and subsequent dysregulation of S1P signaling on astrocytes might also play an important role in disease reactivation after FGL discontinuation.^20^ Whether the extent of astrocytic S1P receptor overexpression is dependent on FGL treatment duration is unclear. In this study, there was no difference in treatment duration between pwMS with and without RDA. In pwMS with sRDA, however, we observed a trend to longer FGL treatment duration compared with those with nonsevere RDA (Table 5).

Of the 433 pwMS who started FGL at our center, 54 were lost to follow up. The main limitations of this study are the retrospective design and the lack of standardized protocol. Most clinical decisions (e.g. switch intervals and subsequent DMT selection) were based on the personal judgments of the treating physicians and influenced by disease characteristics before and during FGL treatment. Therefore, we have to assume that statistical analysis in our cohort data are prone to biases, in particular selection bias and immortal time bias. To mitigate these statistical problems, larger cohorts are required. Further limitations of this study are the incompleteness of the MRI and immunological data. As lymphocyte counts after FGL discontinuation were incompletely available and the duration of the treatment-free period until next DMT was variable, comparison of post-FGL lymphocyte reconstitution between active and nonactive pwMS should be interpreted with caution.

Nevertheless, this study provides important hypothesis generating results, which can help design prospective studies and address open questions of FGL discontinuation. Larger and prospective cohort studies like the SMSC will have to validate our independent risk factors for the occurrence of post-FGL RDA and investigate the effects of short switch intervals and optimal DMT sequence after FGL treatment. The question whether older pwMS with stable disease course under FGL can discontinue the treatment without subsequent DMT is also addressed by the ongoing Discontinuation of Disease-Modifying Therapies in Multiple Sclerosis (DISCO-MS) trial.^21^

## Declarations

**Ethics approval and consent to participate:** The study was approved by the local ethics committees (Project-ID 2019-02254). Participants signed an informed consent (from the Swiss MS cohort study and/or from hospital general informed consent).

**Consent for publication:** Not applicable.

**Availability of data and materials:** Data are available upon reasonable request from the corresponding author.
